# Top-Down versus Bottom-Up Approaches for σ-Functionals Based on the Approximate Exchange Kernel

**DOI:** 10.1021/acs.jpca.4c05289

**Published:** 2025-01-09

**Authors:** Yannick Lemke, Christian Ochsenfeld

**Affiliations:** † Department of Chemistry, Ludwig-Maximilians-Universität München, Butenandtstr. 5-13, D-81377 Munich, Germany; ‡ Max-Planck-Institute for Solid State Research, Heisenbergstr. 1, D-70569 Stuttgart, Germany

## Abstract

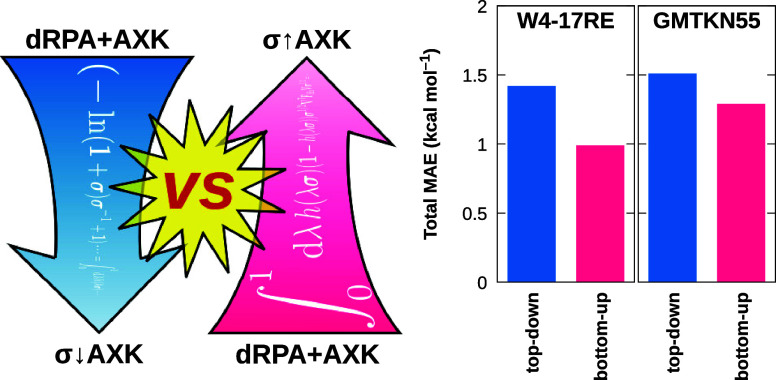

Recently, we investigated a number of so-called σ- and τ-functionals based on the adiabatic-connection fluctuation–dissipation theorem (ACFDT); particularly, extensions of the random phase approximation (RPA) with inclusion of an exchange kernel in the form of an antisymmetrized Hartree kernel. One of these functionals, based upon the approximate exchange kernel (AXK) of Bates and Furche, leads to a nonlinear contribution of the spline function used within σ-functionals, which we previously avoided through the introduction of a simplified “top-down” approach in which the σ-functional modification is inserted a posteriori following the analytic coupling strength integration within the framework of the ACFDT and which was shown to provide excellent performance for the GMTKN55 database when using hybrid PBE0 reference orbitals. In this work, we examine the analytic “bottom-up” approach in which the spline function is inserted a priori, i.e., before evaluation of the analytic coupling strength integral. The new bottom-up functionals, denoted σ↑AXK, considerably improve upon their top-down counterparts for problems dominated by self-interaction and delocalization errors. Despite a small loss of accuracy for noncovalent interactions, the σ↑AXK@PBE0 functionals comprehensively outperform regular σ-functionals, scaled σ-functionals, and the previously derived σ+SOSEX- and τ-functionals in the WTMAD-1 and WTMAD-2 metrics of the GMTKN55 database.

## Introduction

For decades, the search for electron correlation methods which are both highly accurate and computationally feasible has represented one of the focal points of quantum-chemical research. Besides continuous advancements in Kohn–Sham density functional theory (KS-DFT)^1,2^ in the form of new density functional approximations (see ref (3) for a comprehensive review), there has also been a steady interest in methods residing on the fifth and highest rung of “Jacob’s ladder,”^4^ including recent developments of double-hybrid functionals such as the DH23 functional of Becke et al.^5^ as well as methods based on the adiabatic connection fluctuation–dissipation theorem (ACFDT) such as the direct random phase approximation (dRPA). For the latter, concentrated research efforts within the past decade in the groups of Kállay^6^ and Ochsenfeld^7−10^ have led to the development of dRPA and beyond-RPA methods with asymptotically linear scaling behavior, and reduced real-word memory demand through the application of optimized batching schemes.^11^ However, despite promising results for noncovalent interactions^12−14^ and metallic systems^15^ – a central advantage over many-body perturbation theory approaches which fail due to a singularity for vanishing electronic gaps–the accuracy of dRPA has left much to be desired, especially compared to the much more affordable KS-DFT method when combined with empirical dispersion corrections. The use of a long-range dRPA correction on top of range-separated hybrid functionals leads to increased accuracy and faster basis set convergence than the regular dRPA ansatz,^16^ but still does not offer a favorable cost-to-performance ratio compared to dispersion corrected hybrid functionals.

The recent development of σ-functionals in the Görling group^17,18^ as an extension of the dRPA has served to remedy this issue in part, providing substantially better accuracies at virtually identical computational cost as the dRPA. The central idea of σ-functionals is to model the neglected exchange–correlation kernel in the ACFDT by inserting optimized, i.e., empirically fitted, cubic splines into the dRPA energy expression within the spectral representation of the noninteracting density–density response function. A recent study^19^ on the basis set requirements for σ-functionals has concluded that a smaller auxiliary basis set can be chosen to compute the noninteracting response function without a considerable loss of accuracy, which further lowers the computational overhead of σ-functionals, especially when combined with the widely used frozen-core approximation. Further advances of σ-functionals include the computation of molecular properties such as nuclear gradients^20,21^ and, most recently, the analytical computation of nuclear magnetic resonance (NMR) shieldings,^22−24^ for which σ-functionals achieve similar levels of accuracy as the computationally much more demanding coupled-cluster singles and doubles (CCSD) method.^25^

Continuing on the hierarchies of post-Kohn–Sham methods toward the exact correlation energy,^26^ the inclusion of an exchange kernel leads to so-called τ-functionals, for which approximations in the form of a power series approximation^27^ and scaled σ-functionals^28^ have been made. In a previous work,^29^ we investigated the accuracy of τ-functionals obtained from the insertion of cubic splines in a similar fashion to σ-functionals, as well as approximations thereof based on second-order screened exchange (SOSEX)^30^ and the approximate exchange kernel (AXK) of Bates and Furche,^31^ both of which simplify to σ-functionals. In a first full evaluation of σ- and τ-functionals on the widely used GMTKN55 database,^29,32^ we showed that in particular, the AXK-based σ-functionals with reference orbitals obtained from the PBE0 functional^33,34^ – otherwise known as PBEh^33,35^ – perform exceptionally well and provide similar levels of accuracy as the best dispersion-corrected double-hybrid functionals tested in the original GMTKN55 publication,^32^ outperforming both the regular σ-functionals and scaled σ-functionals and thus highlighting the importance of the exchange kernel within the ACFDT energy expression. Despite this high degree of accuracy, however, the σ+AXK-functionals derived in ref (29) contained an additional approximation: To avoid a quadratic term of the inserted cubic splines, we introduced a “top-down” approach in which the σ-functional modification is added *after* carrying out the coupling strength integration and some rearrangement of terms. This approximation holds in the sense that if one does not modify the resulting energy expression through cubic splines, it exactly yields the regular dRPA+AXK energy. In this work, we derive and compare the alternative “bottom-up” approach in which the cubic splines are inserted *before* the coupling strength integration is performed, thus circumventing the approximation made in the “top-down” ansatz.

The manuscript is structured as follows: The Theory section provides a brief summary of the origins of σ-functionals in the context of the adiabatic connection fluctuation–dissipation theorem, and a more detailed derivation of both the top-down approach introduced in ref (29) and the new bottom-up ansatz. The Computational Details section provides an overview of the computational parameters used for the optimization and evaluation of the new σ-functionals, which are then analyzed and discussed in the Results and Discussion section. Finally, we provide some concluding remarks and recommendations in the Conclusion section.

## Theory

Throughout this work, we will consider the working equations for spin-saturated systems with real-valued atomic and molecular orbitals. The analogous equations for spin-polarized systems have been discussed in detail elsewhere, see, e.g., refs (17, 28, and 29).

### Fluctuation–Dissipation Theorem

In the adiabatic connection formalism, the electronic energy for a Slater determinant |Φ⟩ is given by the adiabatic connection fluctuation–dissipation theorem (ACFDT)^36,37^ as
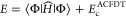
1with the correlation energy
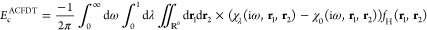
2where
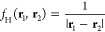
3denotes the Hartree kernel and χ_λ_(iω, **r**_1_, **r**_2_) is the frequency-dependent density–density response function for a given frequency ω and coupling strength λ. The special case λ = 0 represents the noninteracting response function χ_0_(iω, **r**_1_, **r**_2_), for which a closed form in terms of the occupied and virtual molecular orbitals {φ_*i*_} and {φ_*a*_} and their respective orbital energies {ε_*i*_} and {ε_*a*_} is known and reads as
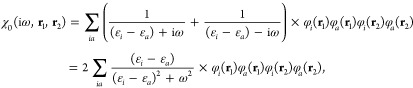
4whereas for the more general case of λ ≠ 0, χ_λ_ is expressed through the Dyson-type equation^38^
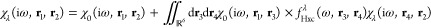
5where
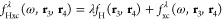
6comprises the Hartree kernel as well as the unknown coupling-strength- and frequency-dependent exchange–correlation kernel *f*_xc_. Note that this formalism assumes a constant density along the path from λ = 0 to λ = 1, which is only strictly valid for local Kohn–Sham potentials. While an adiabatic connection formula for generalized Kohn–Sham theory (GKS), which represents the foundational backbone of hybrid density functional approximations, has been derived,^39^ we would argue that the use of the ACFDT as formulated above is well-established in the context of the random phase approximation, even when hybrid functionals are applied for the generation of reference orbitals.

In typical implementations, the quantities in eqs 2–6 are expressed within the resolution-of-the-identity (RI) approximation,^40−45^ which employs a set of auxiliary basis functions denoted by indices *K*, *L*, ··· to replace the costly four-center-two-electron integrals (4c2e) by simpler 3c2e and 2c2e integrals through so-called “density-fitting” as
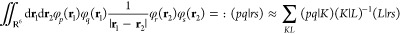
7Defining *C*_*KL*_ = (*K*|*L*)^−1^ and the orthogonalized 3c2e integrals as
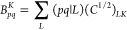
8the correlation energy defined in eq 2 becomes
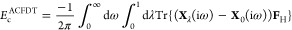
9with **F**_H_ = **1**,
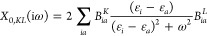
10for the noninteracting response function, and
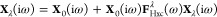
11
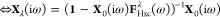
12for the interacting response function.

### Direct Random Phase Approximation and σ-Functionals

Since the exchange–correlation (xc) kernel remains unknown, further approximations must be made. The simplest such approximation comes in the form of the direct random phase approximation (dRPA), in which the xc kernel is neglected entirely, and which forms the basis for σ-functionals. Using the spectral representation of the negative-semidefinite **X**_0_(iω),
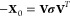
13(the explicit frequency dependence is omitted for the sake of legibility), the dRPA correlation energy is expressed as
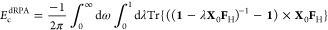
14
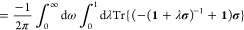
15where in eq 15, we have exploited cyclic permutations of the trace as well as the relations . Since all matrices within the trace are now diagonal, one can define a simple function
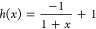
16such that for **σ** = diag(σ_1_, σ_2_, ···), one has
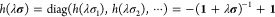
17The idea of σ-functionals^17,18^ is then to model the neglected xc kernel by modifying *h*(*x*), which is realized through the addition of piecewise cubic splines
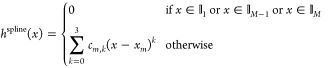
18over *M* intervals
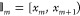
19with *x*_*m*_ < *x*_*m*+1_, *x*_1_ = 0, and *x*_*M*+1_ = *∞*. This ansatz offers vast flexibility, as one is free to choose (i) the underlying density functional approximation for the generation of reference orbitals, (ii) the number and placement of the abscissae {*x*_*m*_}, (iii) the type of splines used to ensure *h*^spline^ is continuous and differentiable on [0, *∞*), and (iv) how to optimize the spline coefficients {*c*_*m*,*k*_}. All of these factors constitute a so-called “parametrization,” of which a considerable number have already been published.^17−19,28,29,46^

### Inclusion of Exchange Kernels and τ-Functionals

Going beyond the direct RPA, one can approximate the exchange–correlation kernel by an exchange kernel that depends linearly on the coupling strength, i.e.,
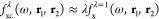
20or equivalently
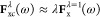
21arriving at what is commonly referred to as “RPA with exchange.” While a variety of approaches which slightly differ in their definitions of *f*_x_ have been published under different names,^27,47−52^ we shall herein adopt the electron–hole exchange kernel discussed in refs (51) and (52), which we will refer to as “RPAx.” The same-spin part of this exchange kernel is given as
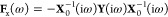
22within the RI representation, with
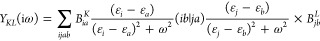
23as discussed, e.g., in refs (10) and (52). Please note that this choice of **F**_x_ merely represents an implementation detail; the following derivations hold for any exchange kernel which can be expressed analytically within the RI approximation.

We note in passing that, given the approximation **F**_Hxc_^λ^(ω) ≈ λ (**F**_H_ + **F**_x_(ω)), one can apply the idea of inserting spectral representations into eqs 9–12 to arrive at so-called τ-functionals,^26^ which have been investigated using a power series approximation^27^ and through cubic splines with either scaled σ values^28^ or exact values of τ obtained from the electron–hole exchange kernel^29^ and generally lead to increased accuracy, albeit at higher computational cost than the regular σ-functionals. In particular, the computation of the matrix **Y** was identified as the most time-consuming step, though efficient strategies to minimize the computational overhead have been developed.^10,53^ Nevertheless, the cost of computing the exchange kernel significantly outweighs the simpler dRPA, σ-functionals, or scaled σ-functionals by 2–3 orders of magnitude,^29^ and thus needs to be taken into consideration for sufficiently large systems.

### Approximate Exchange Kernel

The approximate exchange kernel (AXK) introduced by Bates and Furche^31^ is based on a truncated geometric series expansion of the interacting response function in the RPAx formalism, i.e.,
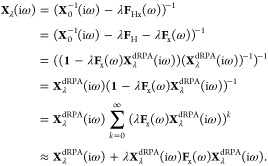
24Inserting this expression into the ACFDT correlation energy, where we once again omit the frequency dependence for the sake of brevity, gives
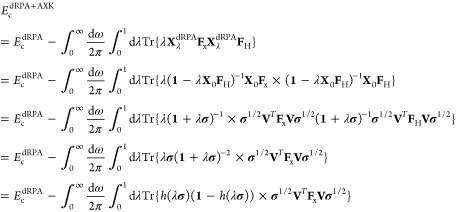
25as we derived in ref (29). Clearly, this expression lends itself to the notion of σ-functionals as we can modify the same function *h*(*x*), though the presence of an *h*^2^(*x*) term in eq 25 makes the expressions slightly more complex than for regular σ-functionals. To avoid this term and achieve only a linear dependence on *h*(*x*), we introduced what we referred to as a “top-down” approach to the correlation energy,^29^ which we denoted as “σ+AXK.” For the remainder of this work, we will refer to these top-down functionals as “σ↓AXK” for better differentiation–please note that the arrow refers to the distinction between the top-down and bottom-up ansatz and is in no way related to the notion of “spin-up” or “spin-down.”

### Top-Down “σ↓AXK” Correlation Energy

For the “top-down” approach, we first carry out the integration over the coupling strength λ in eq 25 analytically to obtain
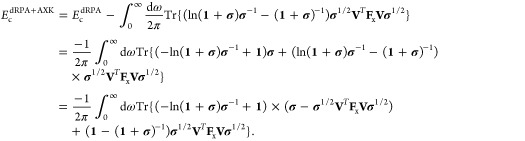
26Next, we realize that the first term in parentheses of eq 26 is precisely equal to the integral of *h*(λ**σ**) over λ, that is,
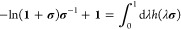
27thereby motivating a reinsertion of the coupling strength integral for just the first term of eq 26 to obtain a σ-functional, i.e.,
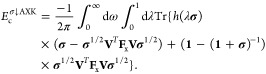
28This reformulation is exact in the sense that if we do not modify the function *h*, the dRPA+AXK energy is recovered exactly. However, if we choose to augment *h* through cubic splines, the top-down approach of eq 28 only represents an approximation to the analytic expression of eq 25.

The motivation for naming this approach “top-down” is as follows: If one pictures the sequence of steps to formulate the dRPA+AXK correlation energy (ACFDT → AXK ) as “going up,” then this approach starts near the top and works downward to transform the dRPA+AXK energy into a σ-functional. In contrast, in the “bottom-up” approach discussed below, the σ-functional is inserted along the path from the bottom upward.

### Bottom-Up “σ↑AXK” Correlation Energy

The new “bottom-up” approach, denoted σ↑AXK, foregoes the approximations of the top-down approach and instead uses the analytic expression of eq 25, in which we insert the spline function before the coupling strength integration is performed. Note that we also augment the dRPA correlation energy in eq 25 through the regular σ-functionals. To that extent, we write for the overall correlation energy
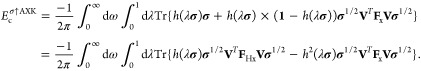
29To evaluate the integrals over λ, we use the following straightforward substitution:
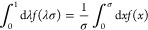
30With the usual decomposition *h*(λσ) = *h*^dRPA^(λσ) + *h*^spline^(λσ), the first term of eq 29 can easily be evaluated as was done in ref (17) to give
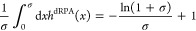
31for the dRPA contribution, whereas the spline contribution is computed as a piecewise integral,
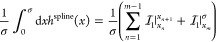
32with
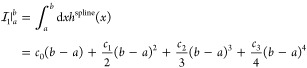
33and spline coefficients *c*_0_, ···, *c*_3_ belonging to the respective interval [*a*, *b*). For the second term of eq 29, we obtain three distinct integrals, namely
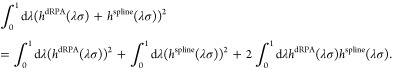
34Using eq 30, the first integral can be evaluated analytically as
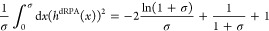
35whereas the second and third integral are once again computed piecewise as
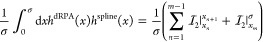
36
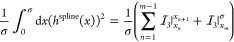
37for σ ∈ [*x*_*m*_, *x*_*m*+1_) and with the auxiliary integrals
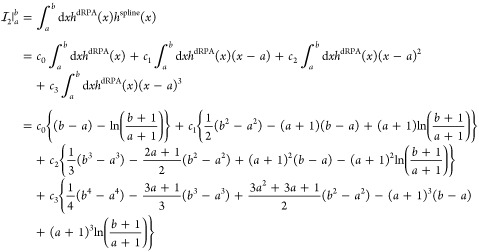
38and
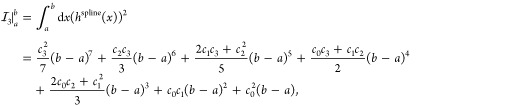
39where *c*_0_, ···, *c*_3_ once again are the spline coefficients belonging to the respective interval [*a*, *b*). These expressions, while somewhat lengthy, are straightforward to implement on top of existing σ-functionals, along with the coefficient derivatives  which are needed for the optimization of the spline coefficients.

As previously mentioned, the computation of the exchange kernel presents the bottleneck in AXK-based calculations. Therefore, the cost of σ↓AXK-functionals and σ↑AXK-functionals is, for all intents and purposes, identical, and a differentiation can be made purely based on their respective accuracies.

## Computational Details

We have implemented the bottom-up σ↑AXK-functionals alongside their top-down variants in our C++ based FermiONs++ program package.^54,55^ For all KS-DFT calculations, we employ the RI-J method^56^ with the def2-universal-jfit auxiliary basis set^57^ in combination with the sn-LinK method^58,59^ on a gm4 grid^60^ for the computation of exact exchange. Exchange–correlation contributions are obtained using the LibXC library^61^ with numerical quadratures being evaluated on a gm5 grid. Throughout this work, we use basis sets of quadruple-ζ quality from the “def2” family (def2-QZVP,^62^ def2-QZVPD,^63^ and def2-QZVPPD^63,64^) in conjunction with their respective auxiliary basis sets^65−67^ for density-fitted integrals within the dRPA+AXK calculation, for which the numerical frequency integration is performed on a 15-point minimax grid first introduced by the Kresse group.^68,69^ Accordingly, we employ a frozen-core approximation and, if applicable, use the def2-ECP effective core potentials^70−73^ to replace core electrons of heavy atoms.

## Results and Discussion

### Optimization of Bottom-Up σ-Functionals

The new σ↑AXK-functionals were optimized on the 16 subsets of the ASCDB database,^74^ which were grouped a posteriori by Morgante and Peverati following a statistical analysis of larger computational chemistry databases.^75^ The optimizations were performed using piecewise cubic Hermite interpolating polynomials (PCHIPs)^76^ and the A1 and A2 parametrizations for σ-functionals introduced in ref (29). The parametrizations are constructed as follows: A fixed set of interval end points *x*_*m*_ (cf. eq 19), taken from the W1 parametrization of ref (18), are distributed logarithmically within the range of 10^–5^ to 10^4/3^ ≈ 21.5 for PBE0 and 10^–5^ to 10^3/2^ ≈ 31.6 for PBE. The precise values of *x*_*m*_ are given in the Supporting Information. The corresponding ordinates *h*_*m*_ ≡ *c*_0,*m*_ are then determined to minimize an objective function, which for the A1 parametrization is the total MAE of the ASCDB database computed from the 16 subsets,
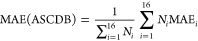
40where *N*_*i*_ and MAE_*i*_ are the number of entries and the computed MAE for subset *i*, respectively. The A2 parametrization aims to minimize the “weighted MAE” (*w*MAE) defined by Peverati as^77^
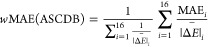
41with  being the average absolute reaction energy of subset *i*. The A1 parametrization is thus weighted more strongly toward reactions with large reaction energies, e.g., atomization reactions and “mindless benchmarks,”^32,78^ whereas the A2 parametrization puts a larger emphasis on reactions with small reaction energies such as noncovalent interactions. The details of the optimization procedure employing the Broyden–Fletcher–Goldfarb–Shanno method^79−82^ have been discussed at length in previous works^17,18,29,46^ and shall therefore not be repeated here. All calculations on the ASCDB database were performed using the def2-QZVPPD basis set; two references from the NLMR11 subset containing the carbon dimer were excluded due to convergence problems in the preliminary SCF.

**Figure 1:**
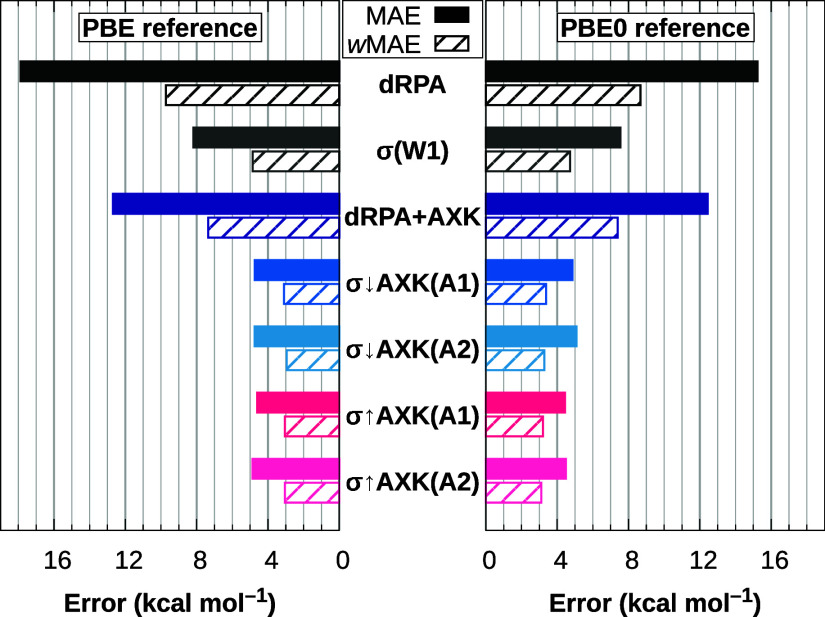
MAE (solid) and *w*MAE (striped) for the ASCDB database using different σ-functionals with PBE and PBE0 reference orbitals.

The resulting total MAE and *w*MAE for σ↑AXK@PBE and σ↑AXK@PBE0 are shown in Figure 1 alongside the previous σ↓AXK functionals, the plain dRPA and dRPA+AXK energies, and the overall best-performing regular σ-functional based on previous testing, σ(W1).^18,29^ The advantages of the σ↓AXK and σ↑AXK approaches over the plain dRPA and dRPA+AXK approaches as well as regular σ-functionals are immediately apparent, whereas the differences between the top-down σ↓AXK and bottom-up σ↑AXK functionals are more nuanced: For the PBE reference, the new bottom-up functionals fall within less than 0.2 kcal mol^–1^ of their top-down counterparts; σ↑AXK(A1)@PBE improves on both the total MAE and *w*MAE compared to σ↓AXK(A1)@PBE (4.61 vs 4.74 kcal mol^–1^ and 3.05 vs 3.10 kcal mol^–1^, respectively), whereas σ↑AXK(A2)@PBE slightly trails its top-down variant with a respective MAE and *w*MAE of 4.87 kcal mol^–1^ and 3.05 kcal mol^–1^ compared to 4.75 kcal mol^–1^ and 2.95 kcal mol^–1^ for σ↓AXK(A2)@PBE. In contrast, we obtain consistent improvements for the σ↑AXK@PBE0 functionals for MAE and *w*MAE regardless of the chosen parametrization. In particular, the respective total MAEs of 4.44 kcal mol^–1^ and 4.49 kcal mol^–1^ for σ↑AXK(A1)@PBE0 and σ↑AXK(A2)@PBE0 correspond to the lowest total MAEs for the ASCDB database of *all* σ-, scaled σ-, and τ-functionals investigated in this work and ref (29). In comparison, σ↓AXK(A1)@PBE0 and σ↓AXK(A2)@PBE0 achieved respective total MAEs of 4.86 kcal mol^–1^ and 5.09 kcal mol^–1^; the improvement of the bottom-up functionals is therefore particularly promising due to the fact that an evaluation of σ↓AXK@PBE0 on the GMTKN55 database^32^ has shown the AXK-based σ-functionals to perform exceptionally well compared to similar methods.^29^

### Evaluation Compared to Top-Down σ-Functionals

To further evaluate the new σ↑AXK functionals, we performed a full evaluation on the 55 subsets of the widely used GMTKN55 database for quantum chemistry,^32^ which are grouped into four categories of chemical reactivity: (i) Basic properties and reaction energies for small systems, (ii) reaction energies for large systems and isomerization reactions, (iii) barrier heights, and (iv) noncovalent interactions, the latter of which is split further into categories centered around inter- and intramolecular noncovalent interactions.

To remain consistent with previous works,^19,28,29^ we replaced the G21EA and G21IP subsets for electron affinities and ionization potentials with their W1-EA and W1-IP counterparts, which replace the reference energies with W1 results from ref (83). For subsets from the “intermolecular noncovalent interactions” category, we employ an averaging scheme between counterpoise-corrected and uncorrected results which we discussed in more detail in ref (29). Again, due to convergence issues, entries within the W4–11 and W4–17 benchmark sets containing either C_2_ or ClOO were excluded, as well as entry 9 of the INV24 subset. The def2-QZVP basis set was used for all calculations with the following exceptions: For benchmark sets featuring anionic systems (AHB21, IL16, W1-EA, and WATER27), we used the augmented def2-QZVPD basis set, whereas the C60ISO and UPU23 subsets were evaluated with the def2-TZVPP basis set.^64,66^

**Figure 2:**
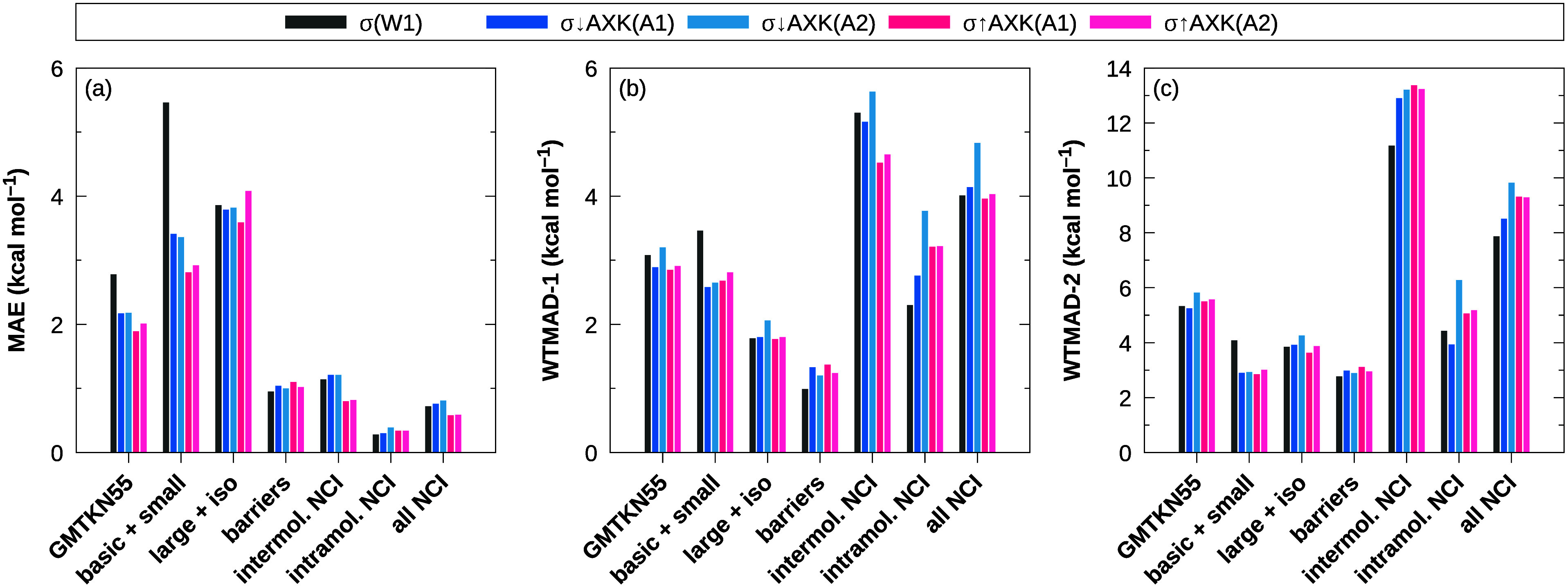
(a) MAE, (b) WTMAD-1, and (c) WTMAD-2 for the GMTKN55 database and its subcategories using different σ-functionals with PBE reference orbitals.

**Figure 3:**
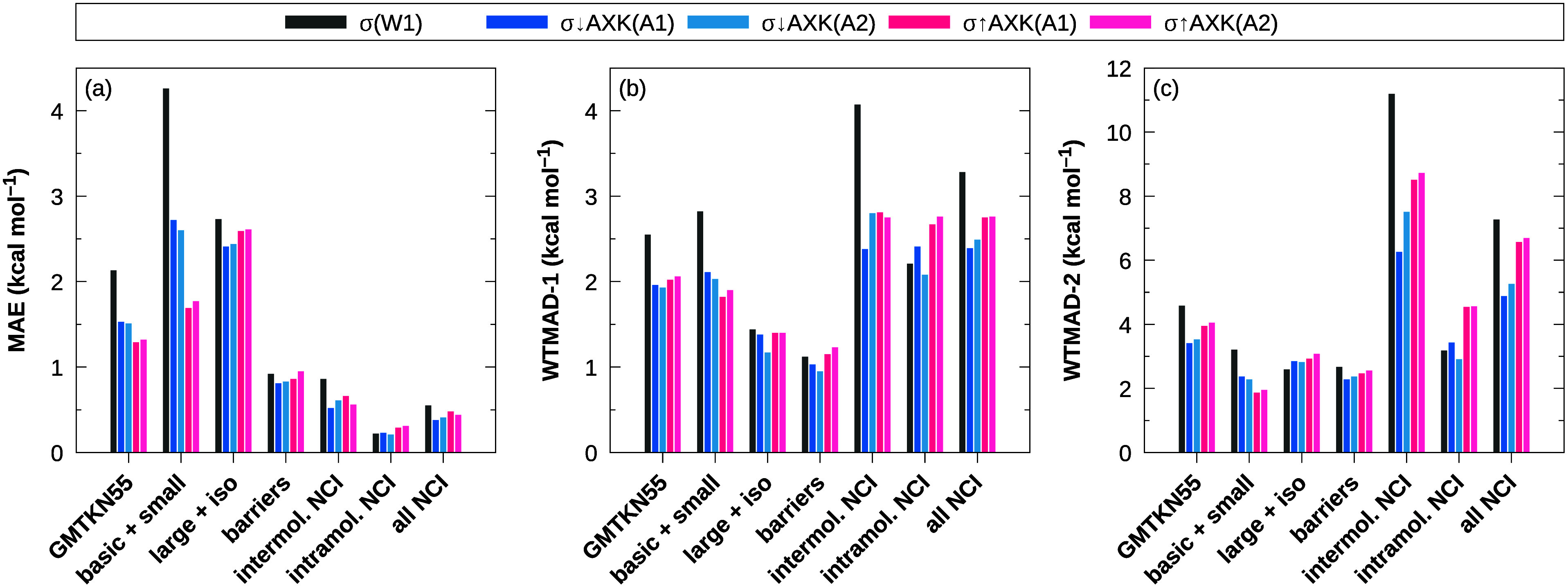
(a) MAE, (b) WTMAD-1, and (c) WTMAD-2 for the GMTKN55 database and its subcategories using different σ-functionals with PBE0 reference orbitals.

The respective MAEs as well as the weighted total mean absolute deviations defined in ref (32) (WTMAD-1 and WTMAD-2) are shown in Figures 2 and 3 for PBE and PBE0 reference orbitals, respectively. In both cases, we observe a significant reduction of the total MAE in the “basic properties” category for the new σ↑AXK-functionals compared to both the regular σ(W1)-functionals as well as the previous σ↓AXK-functionals. Considering the best-performing functionals for each class, for σ↑AXK(A1)@PBE, the total MAE in this category is lowered to 2.81 kcal mol^–1^ from 3.36 kcal mol^–1^ for σ↓AXK(A2)@PBE and 5.46 kcal mol^–1^ for σ(W1)@PBE. Using a PBE0 reference, the best results are also obtained with the σ↑AXK(A1) parametrization at 1.69 kcal mol^–1^, improving by nearly 1 kcal mol^–1^ over σ↓AXK(A2)@PBE0 with a total MAE of 2.60 kcal mol^–1^ and even outperforming the best functional for this category in ref (29), τ(A2)@PBE0, which registered a total MAE of 1.87 kcal mol^–1^.

A more detailed analysis of the subsets within this category (see Tables S3 and S4 in the Supporting Information) reveals that these improvements mainly stem from subsets commonly attributed to self-interaction and delocalization errors, namely those focused on atomization energies, electron and proton affinities, and ionization potentials. We therefore provide a more detailed overview of these subsets, along with the W4–17 benchmark set of atomization energies^84^ and the autogenerated W4–11RE and W4–17RE benchmark sets^84−86^ of reaction energies in Tables 1 and 2. In both cases, there are substantial improvements for the atomization energies of the W4–11 and W4–17 benchmark sets, as well as the ionization potentials and electron affinities of the W1-EA and W1-IP benchmark sets. For the DIPCS10 and PA26 subsets concerned with double ionization potentials, respectively, the trends are less clear-cut, and improvements of the bottom-up σ-functionals over their top-down counterparts (or lack thereof) seem to depend heavily on both the chosen parametrization and the underlying reference orbitals; the same is true for the SIE4 × 4 benchmark set which exposes a different quality of many-electron self-interaction error akin to left–right static correlation in three-electron bonds that are characterized mainly by dynamic correlation.^87,88^

**Table 1:** MAEs in kcal mol^–1^ for Benchmark Sets Characterized by Self-Interaction and Delocalization Errors Using Top-Down and Bottom-Up σ-Functionals and PBE Reference Orbitals

	PBE				
subset	dRPA+AXK	σ↓AXK(A1)	σ↓AXK(A2)	σ↑AXK(A1)	σ↑AXK(A2)
W4–11^a^	14.54	4.11	4.02	2.30	2.63
W4–17^a^	19.62	4.68	4.76	2.93	3.35
W1-EA^b^	3.06	2.46	2.01	1.92	1.72
W1-IP^c^	1.85	5.01	4.74	4.40	3.98
DIPCS10	3.26	8.67	7.55	9.74	8.36
PA26	2.03	4.48	4.34	1.72	1.54
SIE4 × 4	13.41	15.23	15.12	16.83	19.65
W4–11RE^a^	2.25	1.74	1.68	1.54	2.03
W4–17RE^a^	2.02	1.69	1.65	1.51	1.85

a Excluding entries containing C_2_ and ClOO.

b G21EA with W1 reference energies from ref (83).

c G21IP with W1 reference energies from ref (83).

**Table 2:** MAEs in kcal mol^–1^ for Benchmark Sets Characterized by Self-Interaction and Delocalization Errors Using Top-Down and Bottom-Up σ-Functionals and PBE0 Reference Orbitals

	PBE0				
subset	dRPA+AXK	σ↓AXK(A1)	σ↓AXK(A2)	σ↑AXK(A1)	σ↑AXK(A2)
W4–11^a^	15.53	3.48	3.63	1.12	1.22
W4–17^a^	19.22	3.65	4.04	1.25	1.32
W1-EA^b^	4.35	1.59	1.42	1.20	1.07
W1-IP^c^	2.27	3.42	3.13	1.95	2.22
DIPCS10	4.27	3.82	2.15	2.79	3.25
PA26	2.55	2.30	1.72	2.24	1.95
SIE4 × 4	6.42	13.17	13.68	11.81	12.29
W4–11RE^a^	2.74	1.75	1.44	1.00	0.97
W4–17RE^a^	2.52	1.68	1.42	1.02	0.99

a Excluding entries containing C_2_ and ClOO.

b G21EA with W1 reference energies from ref (83).

c G21IP with W1 reference energies from ref (83).

**Figure 4:**
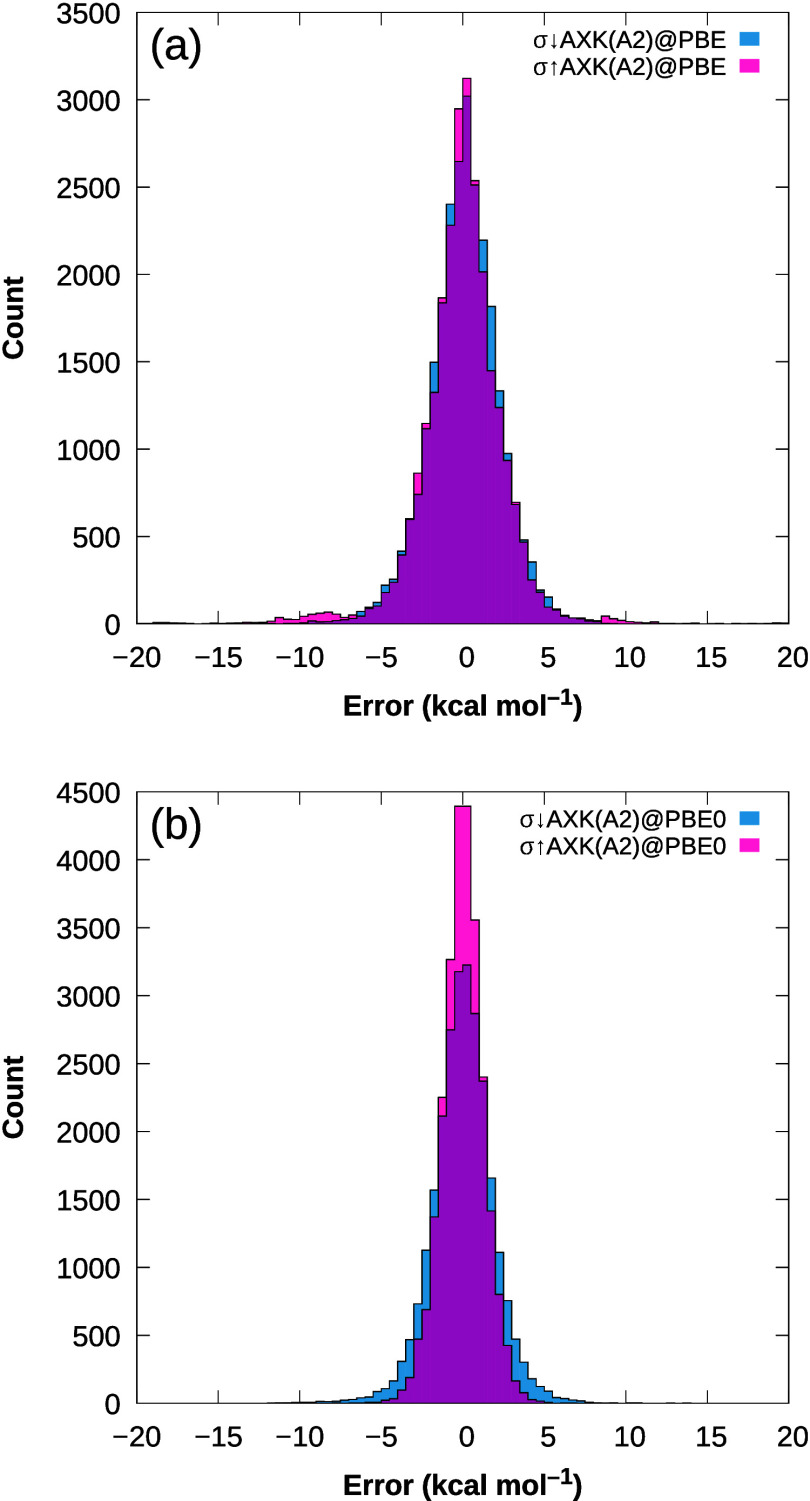
Error distribution for the W4–17RE benchmark set using the top-down σ↓AXK(A2) and bottom-up σ↑AXK(A2) functionals for (a) PBE and (b) PBE0 reference orbitals.

Finally, we consider the reaction energies of the W4–11RE and W4–17RE benchmark sets, which are neither part of our initial optimization nor of the GMTKN55 database, but have so far played an important role in the development and evaluation of σ-functionals due to their large number of data points and high-quality reference energies from W4 theory.^17,18,28,29,46^ While for a PBE reference, we only observe small improvements in the case of σ↑AXK(A1)@PBE, improvements compared to the top-down variant using PBE0 reference orbitals are substantial: The σ↑AXK(A1)@PBE0 and σ↑AXK(A2)@PBE0 functionals achieve respective MAEs of 1.00/0.97 kcal mol^–1^ for the W4–11RE benchmark set and 1.02/0.99 kcal mol^–1^ for the W4–17RE benchmark set, which, to our knowledge, makes σ↑AXK(A2)@PBE0 the first σ-functional shown to produce MAEs of below 1 kcal mol^–1^ for these two benchmark sets using the def2-QZVP basis set. These trends are also reflected in the error distributions for the W4–17RE subset, which we illustrate in Figure 4 for the A2 parametrizations using both PBE and PBE0 reference orbitals. For σ↑AXK(A2)@PBE0, the distribution is considerably narrower than for σ↓AXK(A2)@PBE0, which aligns with the overall lower MAE. Interestingly, the higher MAE of σ↑AXK(A2)@PBE compared to σ↓AXK(A2)@PBE does not appear to originate from a systemic issue which would manifest itself in the form of a broader error distribution. In fact, the error distributions for σ↑AXK(A2)@PBE and σ↓AXK(A2)@PBE nearly coincide, however, the bottom-up variant features a small number of outliers at comparatively large errors of ±10 kcal mol^–1^ which lead to the overall larger MAE.

The substantial improvement for the total MAE for the “basic properties” category also carries over to the total MAE of the entire GMTKN55 database, where all bottom-up σ↑AXK variants outperform their top-down counterparts, even though we do observe a noticeable increase of the total MAE for the “large systems and isomerization reactions” category for σ↑AXK(A2)@PBE, σ↑AXK(A1)@PBE0, and σ↑AXK(A2)@PBE0 which is caused predominantly by higher errors for the challenging MB16–43 and C60ISO benchmark sets (cf. Tables S3 and S4 in the Supporting Information) with comparatively large average reaction energies of 414.73 and 98.25 kcal mol^–1^, respectively. For the weighted WTMAD-1 and WTMAD-2 metrics, the trend of overall improvement for the full GMTKN55 database does not appear to hold, as no σ↑AXK functional consistently outperforms all σ↓AXK functionals. The reason for this lies mostly in a slight deterioration of the accuracy for the remaining categories, in particular, noncovalent interactions. While for the PBE0 reference, total MAEs for noncovalent interactions are increased by less than 0.15 kcal mol^–1^ [Figure 3a] and for the PBE reference, we even observe improvements of the total MAE [Figure 2a], their comparatively large weights in the WTMAD-1 and WTMAD-2 metrics have a disproportionate impact on the overall results. This is especially true for the PBE reference: Whereas the total MAE of the σ↑AXK(A1)@PBE functional for intermolecular noncovalent interactions is substantially reduced compared to both top-down variants (0.80 vs 1.21 kcal mol^–1^), the WTMAD-2 metric is worse by nearly 0.5 kcal mol^–1^ (13.37 vs 12.90 kcal mol^–1^ for σ↑AXK(A1)@PBE and σ↓AXK(A1)@PBE, respectively). This observation suggests that the bottom-up σ↑AXK-functionals predominantly perform worse than their top-down counterparts for subsets with particularly small average reaction energies, which therefore are assigned much larger weights in the computation of WTMAD-1 and WTMAD-2. Furthermore, it should be stressed that σ↓AXK-functionals ranked among the best performing functionals for noncovalent interactions in ref (29), especially those with a PBE0 reference, and both σ↑AXK(A1)@PBE0 and σ↑AXK(A2)@PBE0 still outperform *all* regular σ-functionals, scaled σ-functionals, σ+SOSEX-functionals, and τ-functionals investigated therein in terms of the WTMAD-1 and WTMAD-2 metrics for the full GMTKN55 database. Please note that these findings by no means imply that σ↑AXK-functionals are a universally better choice than, e.g., scaled σ-functionals (even if one were to ignore the important aspect of computational expense), since the vast number of degrees of freedom for both the parametrization and evaluation make a comprehensive ranking of functionals all but impossible. Nevertheless, within the confines of the same parametrizations and basis sets, there are noticeable improvements of our σ↑AXK(A1) and σ↑AXK(A2) functionals compared to, for instance, scσ(A1) and scσ(A2) from ref (29) for the considered benchmark sets. We therefore think the σ↑AXK functionals to present an excellent compromise for general-purpose computations of reaction energies and barrier heights, including systems for which regular σ-functionals and σ↓AXK-functionals suffer from delocalization errors.

### Physical Interpretation of Top-Down versus Bottom-Up Approaches

For a better physical understanding of the differences between the top-down and bottom-up approaches, we herein consider the contributions of the Hartree and exchange kernels within our parametrizations. As discussed by Chen et al.,^53^ the AXK correction exhibits stronger screening compared to the Hartree kernel in the bare dRPA, especially at high coupling strengths λ and large eigenvalues σ, corresponding to long-range electronic separation. Consequently, the AXK correction is predominantly short-ranged in nature, and cancels the self-interaction of same-spin electrons exactly up to second order and approximately in higher orders.^53^

**Figure 5:**
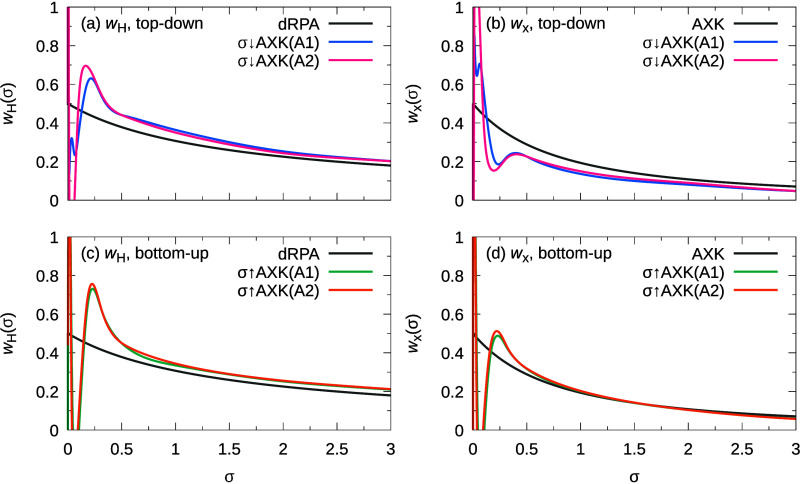
Weight functions of the Hartree (a, c) and exchange (b, d) kernels for σ↓AXK-functionals (a, b) and σ↑AXK-functionals (c, d) for PBE0 reference orbitals.

The insertion of spline functions for the generalization to σ-functionals has an obvious but nontrivial effect on the balance between Hartree and exchange contributions, which we wish to investigate in further detail. To that extent, we consider the coupling-strength-averaged weights of the Hartree and exchange kernels as a function of σ arising from eqs 28 and 29,
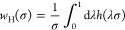
42
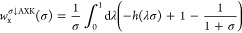
43
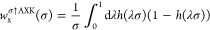
44where the function *h* is partitioned into dRPA and σ-functional contributions, *h*(λσ) = *h*^dRPA^(λσ) + *h*^spline^(λσ). Using these definitions, the correlation energy can be recast as
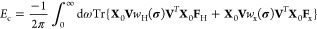
45in analogy to ref (53). Figure 5 shows the functions *w*_H_ and *w*_x_ for the PBE0-parametrized σ↓AXK- and σ↑AXK-functionals, respectively; analogous plots for PBE reference orbitals can be found in the Supporting Information. A number of crucial observations are immediately apparent: First, due to the spline functions, neither *w*_H_ nor *w*_x_ are strictly positive at small values of σ compared to the unmodified dRPA+AXK ansatz; in fact, we observe strong undulations as σ approaches zero. However, it should be stressed that since the spline coefficients of the first and last intervals vanish by definition, the curves coincide in the limits, i.e., we have
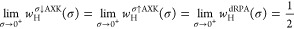
46
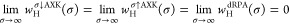
47
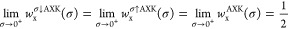
48
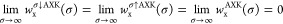
49and that the large magnitude of the oscillations originates from the prefactor if 1/σ in eqs 42–44, which is canceled by the multiplication with **X**_0_ in eq 45. While the weights of the Hartree kernel *w*_H_ behave similarly for the top-down and bottom-up approaches, the same is not true for *w*_x_: Whereas for the σ↑AXK-functionals [Figure 5c,d], the shape of *w*_x_ resembles that of *w*_H_, the definition of the top-down correlation energy in eq 28 means that the leading *h*(λσ) term of *w*_x_ is negated and thus, for σ↓AXK-functionals, *w*_x_ [Figure 5b] behaves opposite to *w*_H_ [Figure 5a]. In particular, this behavior implies that in regions in which the σ-functionals amplify the contributions of **F**_H_, e.g., σ ∼ 0.3, the exchange kernel is more strongly attenuated in σ↓AXK-functionals than in σ↑AXK-functionals, leading to higher contributions of same-spin self-correlation. This distinction likely explains the much better performance of σ↑AXK-functionals for problems dominated by self-interaction errors. Based on these findings, one might consider optimizing two separate sets of splines for the Hartree and exchange kernel, though some care would need to be taken to avoid the double-counting of (approximate) exchange contributions. However, such considerations, while interesting, exceed the scope of this work and have thus not been studied yet.

The severity of unphysical self-correlation can be estimated by considering hydrogen-like systems, for which the correlation energy should be identically equal to zero. In Table 3, we list correlation energies for the hydrogen atom computed with the aug-cc-pV6Z basis set.^89^ As already discussed by Erhard et al.,^28^ the scaled σ-functionals reduce self-correlation by a factor of 10 for the H atom compared to the dRPA and plain σ-functionals (improvements for He^+^ or  are less pronounced, but still substantial). The inclusion of AXK appears to have a similar effect and drastically reduces the correlation energy–an improvement which is, in large part, undone by the σ↓AXK approach, which results in higher correlation energies than even dRPA for PBE reference orbitals and only modest improvements over dRPA for PBE0 reference orbitals. In contrast, the new σ↑AXK ansatz exhibits less unphysical self-correlation, which aligns with our observation that σ↑AXK-functionals are better suited for systems dominated by self-interaction errors, although the very low correlation energies of dRPA+AXK and scaled σ-functionals are not quite matched.

**Table 3:** Correlation Energies for the Hydrogen Atom in m*E*_h_ Computed Using the aug-cc-pV6Z Basis Set

	@PBE	@PBE0
dRPA	–20.768	–18.714
σ(W1)	–23.399	–16.080
scσ(S1)	–2.110	–1.512
dRPA+AXK	–2.219	–1.737
σ↓AXK(A1)	–22.174	–9.121
σ↓AXK(A2)	–24.143	–10.391
σ↑AXK(A1)	–8.851	–5.595
σ↑AXK(A2)	–7.602	–5.540

Finally, we gauge the quality of the predicted dispersion interactions on the neon dimer, which serves as a good model system for pure dispersive interactions without covalent or electrostatic contributions. As the well depth of this system is comparatively small, a high degree of accuracy with respect to integration grids and basis sets is paramount. Therefore, we performed a complete basis set (CBS) extrapolation to account for the basis set incompleteness error (BSIE) using the two-point formula of Helgaker et al.,^90,91^
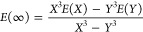
50where we extrapolate using results computed with the aug-cc-pwCVQZ and aug-cc-pwCV5Z basis sets,^92−94^ i.e., *X* = 5, *Y* = 4, with the appropriate auxiliary basis for the computation of the Coulomb and correlation energies.^65,95^ In accordance with the basis sets, we correlate all electrons and do not apply a frozen-core approximation for these calculations. DFT and sn-LinK integrations were performed on the large gm7 grids, whereas the frequency integration used a 50-point Gauss–Legendre grid. To eliminate potential basis set superposition errors, we determined the equilibrium distance and dissociation energy based on counterpoise-corrected interaction energies.^96^

In Table 4, we report the equilibrium bond lengths *R*_e_ and dissociation energies *D*_e_ computed using PBE reference orbitals–an equivalent analysis with PBE0 reference orbitals could unfortunately not be performed due to some numerical instabilities in the  term (eq 38) arising for the quintuple-zeta basis set. While these instabilities are small in absolute terms (≲ 1 μ*E*_h_), they are substantial enough to prohibit a quantitative analysis for weakly bound systems such as Ne_2_. We also list corrected CCSD(T) values from ref (97) in Table 4. A direct comparison reveals that all RPA-based methods significantly underestimate the well depth, whereas at the KS-DFT level, the dissociation energy is greatly overestimated. Note that at the KS-DFT level, we do *not* include any empirical dispersion correction, meaning that the observed interaction potential is highly functional-dependent: Whereas PBE or, for example, functionals including PW91 exchange^98,99^ tend to predict a bound noble gas dimers (albeit not to quantitative accuracy), functionals built upon the exchange functional of Becke^100^ often predict repulsive interactions.^101^ Returning to Table 4, most correlated approaches overestimate the equilibrium bond length; in particular, dRPA@PBE and σ↓AXK(A2)@PBE deviate from the CCSD(T) value by more than 0.1 Å. While the scσ(S1) functional does predict a considerably higher dissociation energy, the bond length is practically unchanged compared to dRPA. In contrast, both σ↑AXK-functionals yield substantially shorter bond lengths which are in much better agreement with the CCSD(T) values. However, while the predicted dissociation energies are increased compared to the plain dRPA+AXK results, the σ-functionals still fall short of the reference value of 28.868 cm^–1^ by a considerable margin.

**Table 4:** Dissociation Energies *D*_e_ and Equilibrium Bond Lengths *R*_e_ for the Neon Dimer Computed from CBS-Extrapolated Interaction Energies^a^ Using PBE Reference Orbitals

	ref^b^	DFT	dRPA	σ	scσ	AXK	σ↓AXK		σ↑AXK	
				W1	S1		A1	A2	A1	A2
*D*_e_ (cm^–1^)	28.868	44.050	11.087	11.669	18.607	11.851	13.296	10.695	19.054	16.614
*R*_e_ (Å)	3.1007	3.0748	3.2029	3.1598	3.1927	3.1895	3.1672	3.2045	3.0790	3.0875

a Counterpoise-corrected interaction energies with CBS extrapolation from aug-cc-pwCVQZ to aug-cc-pwCV5Z basis set according to eq 50.

b CCSD(T)/aug-cc-pV5Z+bf interaction energies with corrections for core correlation, BSIE, full-CI extrapolation, and relativistic effects. Data from ref (97).

**Figure 6:**
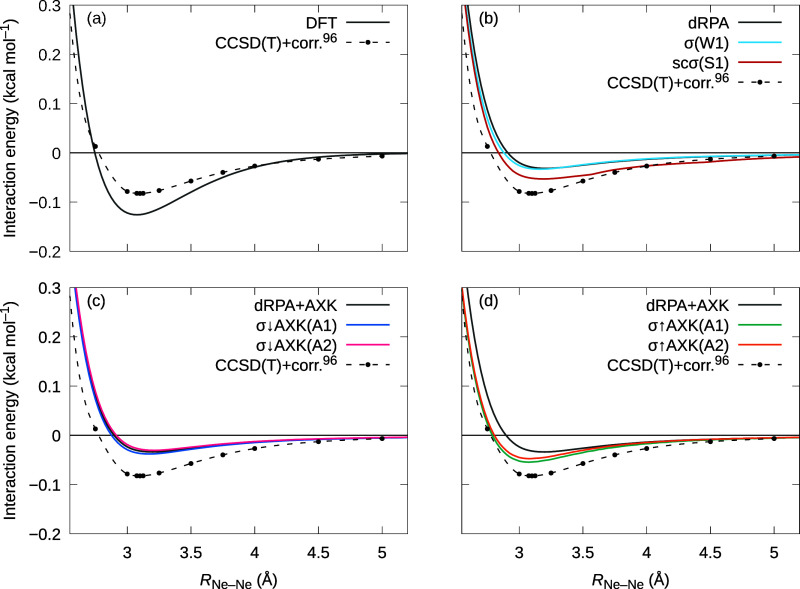
CBS-extrapolated neon dimer interaction energies computed using PBE reference orbitals for (a) KS-DFT, (b) σ-functionals and scaled σ-functionals, (c) top-down σ↓AXK-functionals, and (d) bottom-up σ↑AXK-functionals. For reference, corrected CCSD(T) values from ref (97) are also included; data between points has been interpolated using cubic splines.

In addition, Figure 6 shows the complete dissociation curves for the considered functionals. As noted by Modrzejewski et al.,^14^ at the DFT level the binding energy decays too quickly due to lacking long-range correlation, which is generally corrected for by the RPA-based approaches with the exception of the scσ(S1)-functional, for which the interaction instead appears to decay too slowly compared to CCSD(T). Again, there is a clearly visible difference between the σ↓AXK-functionals, which more or less yield the same dissociation profile as the plain dRPA+AXK method, and the σ↑AXK-functionals, which shift the minimum toward lower bond lengths and a higher binding energy.

It is interesting to note that the σ↑AXK-functionals show better agreement with the corrected CCSD(T) values than σ↓AXK even though they performed, on average, worse for the noncovalent interaction benchmarks of the GMTKN55 database; particularly for the RG18 test set, which includes noble gas oligomers. A potential explanation of this discrepancy lies in the basis set incompleteness of the def2-QZVP basis set used for the evaluation of the GMTKN55 database, as well as the smaller minimax grid we used for the numerical frequency integration. The slow basis set convergence of RPA-based methods for dispersion interactions is a well-documented issue^14,102^ that warrants further investigation, however, an in-depth analysis of individual benchmarks clearly exceeds the scope of this work.

## Conclusions

We have derived and benchmarked so-called “bottom-up” σ↑AXK functionals in which the σ-functional modification is inserted *before* the coupling strength integration is performed, leading to nonlinear terms of the cubic splines. Following optimization on the ASCDB database, we have shown the new σ↑AXK functionals to drastically improve on the previous σ↓AXK functionals for benchmarks commonly connected to electronic self-interaction problems, such as atomization energies, electron affinities, and ionization potentials, while otherwise mostly retaining the excellent performance for the GMTKN55 database without the need for empirical dispersion corrections, particularly when using the hybrid PBE0 functional as reference. Since the computational cost is dominated by the exchange kernel, the use of hybrid functionals for the generation of reference orbitals is highly encouraged; furthermore, the A1 parametrization gives, on average, slightly better overall results than the A2 parametrization. Concerning the distinction between σ↓AXK and σ↑AXK, the precise ordering of the different methods depends on the error metric in question, as σ↓AXK functionals lead to slightly lower errors for noncovalent interactions which are weighted strongly in the WTMAD-1 and WTMAD-2 metrics. We therefore propose the usage of σ↓AXK@PBE0 for systems characterized predominantly by noncovalent interactions, whereas the σ↑AXK@PBE0 functionals seem well-suited for self-interaction-dominated problems, in particular, processes such as ionization which do not retain the particle number. These improvements may be connected to a stronger screening of the exchange kernel for small values of σ in the top-down approach which is remedied in the new bottom-up approach.

While the exploration of different parametrizations, further comparison to existing functionals, and especially the reduction of the large computational overhead arising from the exchange kernel are of course desirable, we expect the σ↑AXK functionals to be a useful supplement to the already existing σ-, scaled σ-, and τ-functionals for the high-quality simulation of chemical reactivity, and a viable alternative to other fifth-rung methods such as dispersion-corrected double-hybrid functionals.

## Supporting Information Available

MAEs for all subsets of the ASCDB and GMTKN55 databases for σ↑AXK functionals; weight functions *w*_H_ and *w*_x_ for PBE reference orbitals; and spline coefficients for σ↑AXK functionals (PDF)
