# Supplementary material for: Top-Down versus Bottom-Up Approaches for σ-Functionals Based on the Approximate Exchange Kernel
Source: J Phys Chem A. 2025 Jan 9;129(3):774–87. doi: 10.1021/acs.jpca.4c05289 (PMC11770766; doi:10.1021/acs.jpca.4c05289)
Supplement: Supplementary file 1 — jp4c05289_si_001.pdf [file jp4c05289_si_001.pdf]

Supporting Information: Top-Down Versus Bottom-Up Approaches for σ-Functionals Based on the Approximate Exchange Kernel

Yannick Lemke[†] and Christian Ochsenfeld[*,†,‡]

†*Chair of Theoretical Chemistry, Department of Chemistry, Ludwig-Maximilians-Universität München, Butenandtstr. 5–13, D-81377 Munich, Germany*

‡*Max-Planck-Institute for Solid State Research, Heisenbergstr. 1, D-70569 Stuttgart, Germany*

E-mail: christian.ochsenfeld@uni-muenchen.de

Contents

1 MAEs of $\sigma\downarrow$AXK- and $\sigma\uparrow$AXK-Functionals for ASCDB **S3**

2 MAEs of $\sigma\downarrow$AXK- and $\sigma\uparrow$AXK-Functionals for GMTKN55 **S5**

3 Hartree and Exchange Contributions for PBE Reference Orbitals **S10**

4 Spline Coefficients for $\sigma\uparrow$AXK@PBE Functionals **S11**

5 Spline Coefficients for $\sigma\uparrow$AXK@PBE0 Functionals **S13**

1 MAEs of $\sigma\downarrow$AXK- and $\sigma\uparrow$AXK-Functionals for ASCDB

Table S1: MAEs in kcal mol^{-1} of $\sigma\downarrow$AXK- and $\sigma\uparrow$AXK-functionals for the ASCDB database using a PBE reference and the def2-QZVPPD basis set. See main text for more computational details.

	PBE				
subset	dRPA+AXK	$\sigma\downarrow$AXK(A1)	$\sigma\downarrow$AXK(A2)	$\sigma\uparrow$AXK(A1)	$\sigma\uparrow$AXK(A2)
AE18pE	9.05	8.06	8.21	7.61	7.75
AR5	77.63	2.50	4.06	1.89	1.80
ARE19	10.44	6.34	6.16	6.48	6.54
BHE20	8.16	2.12	2.20	1.04	1.34
BHH16	6.43	3.58	3.51	3.66	4.16
MBH5	28.97	0.85	0.65	3.79	0.47
MBMH8	24.22	5.62	6.24	4.27	4.92
MBVH7	39.54	9.25	9.54	11.50	12.75
NCC21	4.10	3.27	2.82	2.99	2.86
NCE15	0.39	0.36	0.35	0.21	0.21
NCH21	3.19	2.28	2.14	1.47	1.69
NCW3	19.05	2.34	1.34	1.93	2.43
NLME15	6.17	4.42	5.07	4.85	7.19
NLMH7	24.23	6.18	5.70	2.12	2.56
NLMR11[a]	33.81	16.01	16.35	17.43	17.25
NLSIE9	11.09	7.16	6.47	9.21	8.86
total MAE	12.69	4.74	4.75	4.61	4.87
wMAE	7.35	3.10	2.95	3.05	3.05

[a]Excluding entries 1 and 11.

Table S2: MAEs in kcal mol^{-1} of $\sigma\downarrow$AXK- and $\sigma\uparrow$AXK-functionals for the ASCDB database using a PBE0 reference and the def2-QZVPPD basis set. See main text for more computational details.

	PBE0				
subset	dRPA+AXK	$\sigma\downarrow$AXK(A1)	$\sigma\downarrow$AXK(A2)	$\sigma\uparrow$AXK(A1)	$\sigma\uparrow$AXK(A2)
AE18pE	9.84	8.62	8.98	9.00	8.75
AR5	66.13	1.48	7.38	2.39	2.64
ARE19	11.11	5.87	6.25	5.44	5.52
BHE20	8.76	1.95	2.04	0.93	0.94
BHH16	5.93	3.13	2.13	2.81	2.66
MBH5	40.21	3.15	6.92	2.06	2.67
MBMH8	15.10	3.89	5.48	3.72	3.84
MBVH7	27.00	9.56	10.85	8.13	9.67
NCC21	6.32	5.83	5.20	5.60	5.61
NCE15	0.30	0.21	0.21	0.26	0.18
NCH21	3.25	1.70	1.74	0.66	0.72
NCW3	12.00	0.30	0.16	4.05	0.18
NLME15	9.42	3.99	4.31	3.84	3.98
NLMH7	25.60	5.51	5.86	1.65	1.98
NLMR11[a]	37.61	18.37	16.61	19.05	19.32
NLSIE9	6.88	6.59	6.35	6.00	6.36
total MAE	12.44	4.86	5.09	4.44	4.49
wMAE	7.39	3.38	3.28	3.20	3.11

[a]Excluding entries 1 and 11.

2 MAEs of $\sigma\downarrow$AXK- and $\sigma\uparrow$AXK-Functionals for GMTKN55

Table S3: MAEs in kcal mol^{-1} of $\sigma\downarrow$AXK- and $\sigma\uparrow$AXK-functionals for the GMTKN55 database using a PBE reference and the def2-QZVP basis set. See main text for more computational details.

	PBE				
subset	dRPA+AXK	$\sigma\downarrow$AXK(A1)	$\sigma\downarrow$AXK(A2)	$\sigma\uparrow$AXK(A1)	$\sigma\uparrow$AXK(A2)
**basic properties and reaction energies for small systems**					
W4-11[a]	14.54	4.11	4.02	2.30	2.63
W1-EA[b]	3.06	2.46	2.01	1.92	1.72
W1-IP	1.85	5.01	4.74	4.40	3.98
DIPCS10	3.26	8.67	7.55	9.74	8.36
PA26	2.03	4.48	4.34	1.72	1.54
SIE4x4	13.41	15.23	15.12	16.83	19.65
ALKBDE10	11.61	3.08	3.25	3.17	3.86
YBDE18	2.92	1.04	0.82	1.19	1.06
AL2X6	2.33	0.89	1.19	0.61	0.78
HEAVYSB11	4.33	4.43	4.46	5.02	4.90
NBPRC	1.87	0.74	1.01	0.91	0.91
ALK8	3.18	3.90	4.28	3.69	2.92
RC21	2.55	1.39	1.51	1.33	1.49
G2RC	2.49	1.20	1.40	1.70	1.56
BH76RC	2.64	1.60	1.82	1.54	1.58
FH51	0.98	0.83	0.86	1.08	1.01
TAUT15	0.34	0.68	0.68	0.61	0.61
DC13	3.82	3.46	3.93	4.20	4.77
total MAE	6.43	3.41	3.36	2.81	2.92
WTMAD-1	2.80	2.58	2.65	2.68	2.81
WTMAD-2	3.74	2.90	2.93	2.85	3.01
**reaction energies for large systems and isomerization reactions**					
MB16-43	29.58	6.52	6.91	7.87	8.03
DARC	0.89	1.86	0.74	1.33	1.34
RSE43	0.71	0.39	0.43	0.40	0.45
BSR36	0.79	0.95	1.42	0.80	0.49
CDIE20	0.26	0.45	0.52	0.36	0.39
ISO34	0.35	0.49	0.53	0.64	0.59
ISOL24	0.69	2.47	2.00	1.55	1.80
C60ISO[c]	2.78	11.10	10.93	9.57	11.92
PArel	0.38	0.37	0.65	0.59	0.57
total MAE	5.27	3.79	3.82	3.59	4.08

(continued on next page)

Table S3: (continued)

	PBE				
subset	dRPA+AXK	$\sigma\downarrow$AXK(A1)	$\sigma\downarrow$AXK(A2)	$\sigma\uparrow$AXK(A1)	$\sigma\uparrow$AXK(A2)
WTMAD-1	1.45	1.80	2.06	1.77	1.80
WTMAD-2	2.97	3.92	4.26	3.63	3.87
**reaction barrier heights**					
BH76	2.09	1.39	1.50	1.43	1.47
BHPERI	0.92	1.19	0.91	1.41	1.01
BHDIV10	1.51	1.06	1.25	0.90	0.84
INV24[d]	0.50	0.75	0.60	0.56	0.73
BHROT27	0.25	0.31	0.27	0.29	0.30
PX13	1.40	1.05	0.87	1.32	0.81
WCPT18	0.75	0.80	0.64	1.09	0.86
total MAE	1.29	1.04	1.00	1.10	1.02
WTMAD-1	1.38	1.33	1.20	1.37	1.24
WTMAD-2	3.66	2.98	2.89	3.11	2.95
**intermolecular non-covalent interactions**[e]					
RG18	0.40	0.58	0.63	0.80	0.74
ADIM6	0.61	0.23	0.33	0.20	0.04
S22	0.66	0.63	0.93	0.44	0.55
S66	0.58	0.47	0.65	0.39	0.42
HEAVY28	0.45	0.67	0.66	0.84	0.83
WATER27[b]	7.28	5.35	4.79	2.10	2.11
CARBHB12	1.61	1.05	1.10	0.77	0.88
PNICO23	1.41	1.00	1.17	0.72	0.87
HAL59	1.15	1.40	1.08	1.17	1.14
AHB21[b]	1.20	0.61	0.70	0.24	0.28
CHB6	0.32	0.54	0.78	0.60	0.53
IL16[b]	1.37	0.83	1.19	0.52	0.60
total MAE	1.45	1.21	1.21	0.80	0.82
WTMAD-1	5.92	5.16	5.63	4.52	4.65
WTMAD-2	11.59	12.90	13.21	13.37	13.23
**intramolecular non-covalent interactions**					
IDISP	0.44	1.84	1.69	1.56	1.43
ICONF	0.12	0.14	0.36	0.15	0.16
ACONF	0.07	0.07	0.18	0.18	0.12
Amino20x4	0.14	0.15	0.22	0.15	0.17
PCONF21	0.19	0.30	0.51	0.32	0.40
MCONF	0.17	0.58	0.48	0.56	0.49
SCONF	0.26	0.16	0.19	0.24	0.19

(continued on next page)

Table S3: (continued)

subset	PBE				
	dRPA+AXK	$\sigma\downarrow$AXK(A1)	$\sigma\downarrow$AXK(A2)	$\sigma\uparrow$AXK(A1)	$\sigma\uparrow$AXK(A2)
UPU23[c]	0.42	0.86	1.02	0.91	1.01
BUT14DIOL	0.26	0.04	0.28	0.22	0.22
total MAE	0.21	0.30	0.39	0.34	0.34
WTMAD-1	1.86	2.76	3.77	3.21	3.22
WTMAD-2	3.55	3.93	6.28	5.06	5.18
**all non-covalent interactions**					
total MAE	0.84	0.76	0.81	0.58	0.59
WTMAD-1	4.18	4.14	4.83	3.96	4.03
WTMAD-2	7.66	8.51	9.82	9.31	9.29
**complete GMTKN55 database**					
total MAE	3.43	2.17	2.18	1.89	2.01
WTMAD-1	2.92	2.89	3.20	2.85	2.91
WTMAD-2	5.08	5.25	5.82	5.50	5.57

[a]Excluding entries 131 and 136. [b]def2-QZVPD basis set. [c]def2-TZVPP basis set.
[d]Excluding entry 9. [e]Averaged counterpoise-corrected and uncorrected energies.

Table S4: MAEs in kcal mol^{-1} of $\sigma\downarrow$AXK- and $\sigma\uparrow$AXK-functionals for the GMTKN55 database using a PBE0 reference and the def2-QZVP basis set. See main text for more computational details.

subset	PBE0				
	dRPA+AXK	$\sigma\downarrow$AXK(A1)	$\sigma\downarrow$AXK(A2)	$\sigma\uparrow$AXK(A1)	$\sigma\uparrow$AXK(A2)
**basic properties and reaction energies for small systems**					
W4-11[a]	15.53	3.48	3.63	1.12	1.22
W1-EA[b]	4.35	1.59	1.42	1.20	1.07
W1-IP	2.27	3.42	3.13	1.95	2.22
DIPCS10	4.27	3.82	2.15	2.79	3.25
PA26	2.55	2.30	1.72	2.24	1.95
SIE4x4	6.42	13.17	13.68	11.81	12.29
ALKBDE10	11.15	3.37	2.57	2.82	2.71
YBDE18	3.38	1.51	1.31	0.73	0.73
AL2X6	2.30	0.98	1.50	1.07	0.89
HEAVYSB11	4.67	2.90	3.44	3.09	3.46
NBPRC	1.47	0.59	0.60	0.59	0.45
ALK8	2.73	3.22	2.68	3.34	3.48
RC21	1.33	1.18	0.97	1.22	1.51

(continued on next page)

Table S4: (continued)

subset	PBE0 dRPA+AXK	$\sigma\downarrow$AXK(A1)	$\sigma\downarrow$AXK(A2)	$\sigma\uparrow$AXK(A1)	$\sigma\uparrow$AXK(A2)
G2RC	2.05	1.84	1.25	1.15	1.22
BH76RC	2.07	1.33	1.41	0.85	0.89
FH51	0.84	0.77	0.72	0.69	0.69
TAUT15	0.30	0.43	0.38	0.36	0.40
DC13	3.58	3.05	2.34	2.25	2.45
total MAE	6.49	2.72	2.60	1.69	1.77
WTMAD-1	2.32	2.11	2.03	1.82	1.90
WTMAD-2	3.25	2.37	2.28	1.87	1.95
**reaction energies for large systems and isomerization reactions**					
MB16-43	22.61	5.77	5.58	5.54	5.97
DARC	0.64	2.17	0.85	2.09	1.96
RSE43	0.69	0.33	0.26	0.29	0.32
BSR36	0.33	1.61	2.08	1.49	1.96
CDIE20	0.17	0.17	0.16	0.21	0.11
ISO34	0.50	0.37	0.41	0.28	0.28
ISOL24	0.80	1.13	0.92	1.55	1.74
C60ISO[c]	2.09	5.11	5.55	6.15	5.63
PArel	0.52	0.40	0.33	0.37	0.41
total MAE	4.07	2.41	2.44	2.59	2.61
WTMAD-1	1.37	1.38	1.17	1.40	1.40
WTMAD-2	2.63	2.85	2.82	2.93	3.08
**reaction barrier heights**					
BH76	2.38	1.14	1.25	1.13	1.12
BHPERI	1.82	0.52	0.74	0.86	1.04
BHDIV10	1.86	0.83	0.78	0.62	0.78
INV24[d]	0.85	0.39	0.32	0.52	0.48
BHROT27	0.10	0.23	0.21	0.29	0.22
PX13	2.13	0.80	0.27	0.97	1.51
WCPT18	1.69	1.23	1.16	1.06	1.46
total MAE	1.69	0.81	0.83	0.86	0.95
WTMAD-1	1.68	1.03	0.95	1.15	1.23
WTMAD-2	4.47	2.28	2.37	2.47	2.56
**intermolecular non-covalent interactions**[e]					
RG18	0.36	0.28	0.35	0.39	0.39
ADIM6	0.86	0.08	0.05	0.08	0.03
S22	0.62	0.21	0.32	0.33	0.28
S66	0.56	0.11	0.17	0.19	0.20

(continued on next page)

Table S4: (continued)

	PBE0				
subset	dRPA+AXK	$\sigma\downarrow$AXK(A1)	$\sigma\downarrow$AXK(A2)	$\sigma\uparrow$AXK(A1)	$\sigma\uparrow$AXK(A2)
HEAVY28	0.40	0.38	0.48	0.63	0.66
WATER27[b]	5.06	2.05	2.09	2.85	1.64
CARBHB12	0.69	0.45	0.46	0.34	0.31
PNICO23	0.96	0.50	0.64	0.41	0.36
HAL59	0.82	0.73	0.74	0.90	1.00
AHB21[b]	0.61	0.28	0.41	0.27	0.23
CHB6	0.35	0.69	0.79	0.46	0.42
IL16[b]	1.26	0.25	0.87	0.25	0.28
total MAE	1.06	0.52	0.61	0.66	0.56
WTMAD-1	4.51	2.38	2.80	2.81	2.75
WTMAD-2	9.29	6.26	7.51	8.51	8.72
**intramolecular non-covalent interactions**					
IDISP	0.76	1.50	0.99	0.87	1.54
ICONF	0.19	0.10	0.17	0.19	0.14
ACONF	0.16	0.08	0.04	0.05	0.05
Amino20x4	0.13	0.10	0.10	0.17	0.15
PCONF21	0.17	0.48	0.23	0.41	0.29
MCONF	0.07	0.39	0.31	0.46	0.52
SCONF	0.24	0.26	0.20	0.19	0.28
UPU23[c]	0.43	0.59	0.66	0.67	0.67
BUT14DIOL	0.16	0.03	0.06	0.17	0.23
total MAE	0.18	0.23	0.21	0.29	0.31
WTMAD-1	1.82	2.41	2.08	2.67	2.76
WTMAD-2	3.10	3.43	2.91	4.54	4.56
**all non-covalent interactions**					
total MAE	0.63	0.38	0.41	0.48	0.44
WTMAD-1	3.36	2.39	2.49	2.75	2.76
WTMAD-2	6.26	4.88	5.26	6.57	6.69
**complete GMTKN55 database**					
total MAE	3.19	1.53	1.51	1.29	1.32
WTMAD-1	2.48	1.96	1.93	2.02	2.06
WTMAD-2	4.44	3.41	3.53	3.95	4.05

[a]Excluding entries 131 and 136. [b]def2-QZVPD basis set. [c]def2-TZVPP basis set.
[d]Excluding entry 9. [e]Averaged counterpoise-corrected and uncorrected energies.

3 Hartree and Exchange Contributions for PBE Reference Orbitals

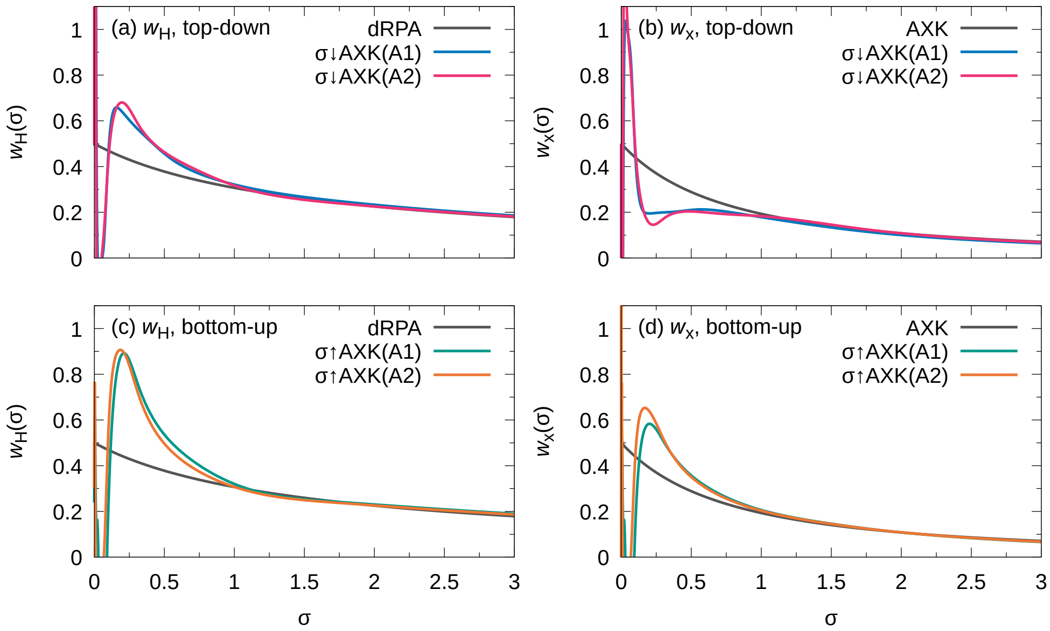

Figure S1: Weight functions of the Hartree ((a) and (c)) and exchange ((b) and (d)) kernels for $\sigma\downarrow$AXK-functionals ((a) and (b)) and $\sigma\uparrow$AXK-functionals ((c) and (d)) for PBE reference orbitals. See main text for definition of w_{H} and w_{x}.

4 Spline Coefficients for $\sigma\!\uparrow$AXK@PBE Functionals

Table S5: Optimized spline coefficients for $\sigma\!\uparrow$AXK(A1)@PBE.

x_m	$c_{m,0}$	$c_{m,1}$	$c_{m,2}$	$c_{m,3}$
0.0000000000e+00	0.0000000000e+00	0.0000000000e+00	0.0000000000e+00	0.0000000000e+00
1.0000000000e-05	0.0000000000e+00	0.0000000000e+00	-2.1251183099e+04	1.5741617110e+08
1.0000000000e-04	-5.7378194366e-05	0.0000000000e+00	1.0156949282e+04	-5.5227898017e+07
2.1544346900e-04	-6.9847513623e-06	1.3700809740e-01	-2.2119023116e+02	-1.4539033278e+05
4.6415888300e-04	1.1171703912e-05	0.0000000000e+00	-4.1479660727e+02	5.1606915322e+05
1.0000000000e-03	-2.8527885197e-05	0.0000000000e+00	1.8336834133e+02	-6.0093451803e+04
1.7782794100e-03	5.4212608816e-05	1.7622440125e-01	1.5913682469e+03	-7.9722317195e+05
3.1622776600e-03	1.2328715002e-03	0.0000000000e+00	-6.4468190750e+02	1.0557483294e+05
5.6234132500e-03	-1.0982249066e-03	-1.2548392157e+00	-8.0187310113e+02	1.4102497545e+05
1.0000000000e-02	-1.0127306663e-02	-1.6998001885e-01	3.1200366243e+01	-2.5347469768e+03
1.7782794100e-02	-1.0755287807e-02	-1.4493116579e-01	-5.4805833317e+02	2.1351050674e+04
3.1622776600e-02	-6.1137798700e-02	-3.0460982375e+00	-6.2693593921e+01	3.3745287668e+03
5.6234132500e-02	-1.2377510090e-01	0.0000000000e+00	3.9659455712e+02	-5.7464413940e+03
1.0000000000e-01	1.5415028156e-01	1.6935262088e+00	-8.8770420731e+00	-1.7156606592e+01
1.7782794100e-01	2.2409608137e-01	0.0000000000e+00	-2.2728640341e+01	1.0225605674e+02
3.1622776600e-01	5.9818945655e-02	-4.1528522882e-01	1.5135983428e+00	-3.1369082972e+00
5.6234132500e-01	2.5294684107e-03	-2.4027641453e-01	-6.1558697147e-01	1.3558343993e+00
1.0000000000e+00	-1.0688079565e-01	0.0000000000e+00	9.2139180893e-01	-7.8925537289e-01
1.7782794100e+00	7.9153996925e-02	0.0000000000e+00	-3.7944898931e-03	1.8277912770e-03
3.1622776600e+00	7.6731276908e-02	0.0000000000e+00	1.5716068956e-02	-4.2571320924e-03
5.6234132500e+00	1.0846300706e-01	0.0000000000e+00	-3.4048160618e-03	2.3219227981e-04
1.5399265300e+01	0.0000000000e+00	0.0000000000e+00	0.0000000000e+00	0.0000000000e+00
3.1622776600e+01	0.0000000000e+00	0.0000000000e+00	0.0000000000e+00	0.0000000000e+00

Table S6: Optimized spline coefficients for $\sigma\uparrow$AXK(A2)@PBE.

x_m	$c_{m,0}$	$c_{m,1}$	$c_{m,2}$	$c_{m,3}$
0.0000000000e+00	0.0000000000e+00	0.0000000000e+00	0.0000000000e+00	0.0000000000e+00
1.0000000000e-05	0.0000000000e+00	0.0000000000e+00	-1.5965380608e+04	1.1826207857e+08
1.0000000000e-04	-4.3106527641e-05	0.0000000000e+00	2.2928731717e+03	-1.1245120383e+07
2.1544346900e-04	-2.9850001016e-05	7.9796745117e-02	-3.2090146915e+02	9.2838409211e+05
4.6415888300e-04	-1.5570541129e-05	9.2458191022e-02	9.8414520777e+02	-6.9475374364e+05
1.0000000000e-03	2.0965526972e-04	5.4870415540e-01	7.2005641364e+02	-4.4706776710e+05
1.7782794100e-03	8.6209613486e-04	8.5712220904e-01	6.7098327136e+02	-4.7236940276e+05
3.1622776600e-03	2.0813451207e-03	0.0000000000e+00	-1.9286252948e+03	3.3397038803e+05
5.6234132500e-03	-4.6220173864e-03	-3.4244520321e+00	-1.1758540316e+03	1.8824114899e+05
1.0000000000e-02	-2.6351822849e-02	-2.8999045764e+00	7.9846264196e-01	1.5890078834e+04
1.7782794100e-02	-4.1381941539e-02	0.0000000000e+00	1.2174585719e+02	-5.8644514033e+03
3.1622776600e-02	-3.3608666776e-02	0.0000000000e+00	-5.9265434678e+01	1.6053682676e+03
5.6234132500e-02	-4.5574730210e-02	0.0000000000e+00	3.2068033159e+02	-4.8079280158e+03
1.0000000000e-01	1.6561753417e-01	4.4165194799e-01	-3.2257170312e+00	3.3266194146e+00
1.7782794100e-01	1.8201985008e-01	0.0000000000e+00	-2.1648263841e+01	9.7556940734e+01
3.1622776600e-01	2.5979531056e-02	-3.8626520710e-01	1.2117835295e+00	-2.1036563697e+00
5.6234132500e-01	-2.7045950096e-02	-1.7205978104e-01	-1.6265702090e-01	5.4719274572e-01
1.0000000000e+00	-8.7633639925e-02	0.0000000000e+00	7.3181017486e-01	-6.2609694834e-01
1.7782794100e+00	6.0483885166e-02	1.3894303545e-03	-1.4675940457e-03	6.6516243228e-04
3.1622776600e+00	6.1359082635e-02	1.1493936220e-03	9.9251328076e-02	-2.6948220509e-02
5.6234132500e+00	2.6363962245e-01	0.0000000000e+00	-8.2760421769e-03	5.6438675862e-04
1.5399265300e+01	0.0000000000e+00	0.0000000000e+00	0.0000000000e+00	0.0000000000e+00
3.1622776600e+01	0.0000000000e+00	0.0000000000e+00	0.0000000000e+00	0.0000000000e+00

5 Spline Coefficients for $\sigma\uparrow$AXK@PBE0 Functionals

Table S7: Optimized spline coefficients for $\sigma\uparrow$AXK(A1)@PBE0.

x_m	$c_{m,0}$	$c_{m,1}$	$c_{m,2}$	$c_{m,3}$
0.0000000000e+00	0.0000000000e+00	0.0000000000e+00	0.0000000000e+00	0.0000000000e+00
1.0000000000e-05	0.0000000000e+00	0.0000000000e+00	-8.0808909891e+04	5.9858451771e+08
1.0000000000e-04	-2.1818405671e-04	0.0000000000e+00	3.6708805382e+00	-5.7119879793e+03
2.1544346900e-04	-2.1814392228e-04	6.1918404230e-04	2.4789543217e+03	-4.7994120127e+06
4.6415888300e-04	-1.3848415749e-04	3.4306185927e-01	-2.4544367831e+02	5.7748738559e+05
1.0000000000e-03	6.3717907812e-05	5.7745864389e-01	1.5649974109e+03	-5.8216504850e+05
1.7782794100e-03	1.1866471221e-03	1.9555841535e+00	2.1798724368e+03	-8.0931754302e+05
3.1622776600e-03	5.9231258790e-03	3.3388387589e+00	2.1892516884e+02	-1.0470441149e+05
5.6234132500e-03	1.3905644271e-02	2.5138047703e+00	2.0666217643e+02	-7.5226048590e+04
1.0000000000e-02	2.2559738388e-02	0.0000000000e+00	-8.1745365674e+01	2.4648921498e+03
1.7782794100e-02	1.8770263344e-02	-8.2450521520e-01	-7.1382690210e+02	2.9604838053e+04
3.1622776600e-02	-5.0889066283e-02	-3.5712224998e+00	-3.2174186099e+01	2.8368074733e+03
5.6234132500e-02	-1.1598032180e-01	0.0000000000e+00	1.2772506576e+02	-1.4586322079e+03
1.0000000000e-01	6.3916501145e-03	2.7981803537e+00	3.6052170316e+01	-4.6280618259e+02
1.7782794100e-01	2.2436765638e-01	0.0000000000e+00	-3.7082983219e+01	1.7861449210e+02
3.1622776600e-01	-1.2435140943e-02	-7.3671493673e-04	1.9150084967e-03	-1.1331119501e-03
5.6234132500e-01	-1.2517352742e-02	0.0000000000e+00	1.1151505602e+00	-1.6208276688e+00
1.0000000000e+00	6.5208067658e-02	4.4725762630e-02	-7.4272371385e-02	6.0408727740e-02
1.7782794100e+00	8.3506825073e-02	3.8888561351e-02	3.2978893245e-01	-2.8302465469e-01
2.6101572200e+00	1.8114743419e-01	0.0000000000e+00	-9.9513738977e-03	8.9775242352e-04
1.0000000000e+01	0.0000000000e+00	0.0000000000e+00	0.0000000000e+00	0.0000000000e+00
2.1544346900e+01	0.0000000000e+00	0.0000000000e+00	0.0000000000e+00	0.0000000000e+00

Table S8: Optimized spline coefficients for $\sigma\uparrow$AXK(A2)@PBE0.

x_m	$c_{m,0}$	$c_{m,1}$	$c_{m,2}$	$c_{m,3}$
0.0000000000e+00	0.0000000000e+00	0.0000000000e+00	0.0000000000e+00	0.0000000000e+00
1.0000000000e-05	0.0000000000e+00	0.0000000000e+00	-4.9943576471e+03	3.6995241831e+07
1.0000000000e-04	-1.3484765647e-05	0.0000000000e+00	1.1594150532e+03	-3.0637187750e+06
2.1544346900e-04	-2.7466621228e-06	1.4520146317e-01	2.9616338101e+03	-5.9625893035e+06
4.6415888300e-04	1.2483526740e-04	5.1188359757e-01	-2.9486838432e+02	5.4550747541e+05
1.0000000000e-03	3.9838762875e-04	6.6576604030e-01	4.1775608890e+02	3.9539529284e+04
1.7782794100e-03	1.1882220290e-03	1.3878774785e+00	1.5589260914e+03	-5.4944451995e+05
3.1622776600e-03	4.6385218052e-03	2.5456770207e+00	3.4050929629e+02	-1.2736364014e+05
5.6234132500e-03	1.1067625726e-02	1.9073594283e+00	1.1480349976e+02	-5.0680042452e+04
1.0000000000e-02	1.7365776715e-02	0.0000000000e+00	-1.6586676745e+01	3.4745686250e+02
1.7782794100e-02	1.6524888049e-02	-1.9504302951e-01	-1.3990347164e+03	6.6904671609e+04
3.1622776600e-02	-7.6789754106e-02	-4.7448584169e-01	1.1290862378e+01	-4.4729786519e+01
5.6234132500e-02	-8.2295218035e-02	0.0000000000e+00	1.0172854647e+02	-1.1230983217e+03
1.0000000000e-01	1.8409960136e-02	2.4507562287e+00	3.9535840121e+01	-4.7352785510e+02
1.7782794100e-01	2.2539351164e-01	0.0000000000e+00	-4.0266565587e+01	1.9396250724e+02
3.1622776600e-01	-3.1701953699e-02	0.0000000000e+00	2.7955440886e+00	-7.1738345309e+00
5.6234132500e-01	3.0685035813e-02	7.2444592520e-02	-1.7681955595e-01	2.2899531101e-01
1.0000000000e+00	4.7719111165e-02	4.9260167480e-02	4.6207112947e-02	-2.1035447777e-02
1.7782794100e+00	1.0412930484e-01	8.2959555603e-02	1.5162105223e-01	-1.6146912842e-01
2.6101572200e+00	1.8511241556e-01	0.0000000000e+00	-1.0169191016e-02	9.1740255905e-04
1.0000000000e+01	0.0000000000e+00	0.0000000000e+00	0.0000000000e+00	0.0000000000e+00
2.1544346900e+01	0.0000000000e+00	0.0000000000e+00	0.0000000000e+00	0.0000000000e+00
